# Liver Enzymes Abnormalities among Highly Active Antiretroviral Therapy Experienced and HAART Naïve HIV-1 Infected Patients at Debre Tabor Hospital, North West Ethiopia: A Comparative Cross-Sectional Study

**DOI:** 10.1155/2016/1985452

**Published:** 2016-07-14

**Authors:** Melashu Balew Shiferaw, Ketema Tafess Tulu, Amtatachew Moges Zegeye, Amarech Asratie Wubante

**Affiliations:** ^1^Bahir Dar Regional Health Research Laboratory Center, Bahir Dar, Ethiopia; ^2^Department of Biomedical Science, School of Health and Hospital, Adama Science and Technology University, Asella, Ethiopia; ^3^North Shoa Zone Health Department, Debre Berhan, Ethiopia; ^4^West Gojjam Zone Health Department, Finote Selam, Ethiopia

## Abstract

Liver disease has emerged as the most common non-AIDS-related cause of death in HIV patients. However, there is limited data regarding this condition including our setting in Ethiopia. Hence, liver enzyme abnormalities among highly active antiretroviral therapy (HAART) experienced and HAART naïve patients were assessed in this study. A total of 164 HAART experienced and 164 HAART naïve patients were studied. Blood specimen was collected to determine alanine aminotransferase (ALT) and aspartate aminotransferase (AST), CD4 count, and viral hepatitis. The prevalence of liver enzyme abnormality was 20.1% and 22.0% among HAART experienced and HAART naïve patients, respectively. The HAART experienced patients had higher mean ALT than HAART naïve patients (*P* = 0.002). Viral hepatitis (AOR = 6.02; 95% CI = 1.87–19.39), opportunistic infections (AOR = 2.91; 95% CI = 1.04–8.19), current CD4 count <200 cells/mm^3^ (AOR = 2.16; 95% CI = 1.06–4.39), and male sex (AOR = 1.83; 95% CI = 1.001–3.33) were associated with elevated ALT and/or AST. In conclusion, liver enzyme abnormalities were high in both HAART experienced and HAART naïve HIV-1 infected patients. Hence, monitoring and management of liver enzyme abnormalities in HIV-1 infected patients are important in our setting.

## 1. Introduction

Liver disease has emerged as the most common non-AIDS-related cause of death among HIV infected patients, accounting for 14–18% of all deaths [1, 2]. Nearly half of deaths among hospitalized HIV infected patients in the HAART era have been attributed to liver disease [3, 4]. It ranges from asymptomatic mild elevations of liver enzymes to cirrhosis and end stage liver disease with all its complications (e.g., ascites, esophageal varices, and hepatic encephalopathy). Liver cirrhosis is a more serious consequence with an estimate overall prevalence of 8.3% in HIV infected persons [5].

Liver disease is often reflected by biochemical abnormalities of liver function. Many authors agree that elevated serum activity of the two commonly used liver enzymes (alanine aminotransferase [ALT] and aspartate aminotransferase [AST]) that are involved in breakdown of amino acids reflects liver cell injury [6–8]. Opportunistic infections, AIDS related neoplasms [9, 10], concomitant infection with chronic hepatitis C virus (HCV), chronic hepatitis B virus (HBV), medication-related hepatotoxicity, alcohol abuse, and nonalcoholic fatty liver disease are some of the factors accounting for liver enzyme abnormalities in people infected with HIV [11–14]. However, the risk factors and the burden of liver disease might be different in different geographical areas including our setting in Ethiopia.

Managing liver disease is an important component of the care of HIV infected individuals. However, there is limited study that evaluated the burden and causes of liver enzyme abnormality among HIV patients in the clinical settings in Ethiopia. The aim of this study was to (1) determine the prevalence of liver enzyme abnormalities and (2) identify factors associated with liver enzyme elevations among HIV-1 infected patients.

## 2. Material and Methods

### 2.1. Study Setting and Study Subjects

This comparative cross-sectional study design was conducted to assess liver enzymes abnormalities in HIV-1 infected patients at Debre Tabor Hospital from February to April 2013. Debre Tabor Hospital provides health services to more than 2.3 million people for inpatients with 88 beds and outpatients. Antiretroviral therapy (ART) service for both HAART experienced and HAART naïve HIV-1 patients has been given in the hospital since 2006. A total of 5453 HIV infected patients had followed their health outcome at this hospital. HAART experienced patients with 1a (d4T+3TC+NVP), 1b (d4T+3TC+ EFV), 1c (AZT+3TC+ NVP), 1d (AZT+3TC+EFV), 1e (TDF+3TC+EFV), and 1f (TDF+3TC+NVP) regimen and HAART naïve HIV infected patients were study subjects. In this study, 164 HAART experienced and 164 HAART naïve patients were included using 30% prevalence of liver enzyme abnormality among HIV infected patients in a similar setting [15], 95% confidence interval, odds ratio of 2, and 80% power. Study subjects were selected consecutively in each group. Patients who had no sufficient blood specimen to determine liver enzymes (ALT and/or AST) level and history of taking some drugs other than HAART and prophylaxis were excluded from this study.

### 2.2. Operational Definitions

*Abnormal Liver Enzymes.* ALT or AST enzyme level >1.25 times upper limit normal value (ULN).

*Severe Hepatotoxicity.* ALT or AST enzyme level >5.1–10X ULN (grade 3) and >10X ULN (grade 4) [16].

*Good Adherence.* HIV-1 patients had taken ≥95% of the prescribed dosage regimen.

### 2.3. Data Collection Procedure and Quality Control

Demographic and clinical data were collected using a structured questionnaire. From each participant, 3–5 mL of venous blood specimen was collected in a plain vacutainer tube and centrifuged at 300 rpm for 10 minute to get sera for liver enzyme determination and viral hepatitis screening. In addition, around 3 mL of venous blood was collected in a vacutainer tube with Ethylenediaminetetraacetic Acid (EDTA) anticoagulant to measure CD4 count using BD FACS count machine (BECTON DICKINSON, USA). Liver enzyme biomarkers (ALT and AST) were determined using 5010 photometer (ROBERT RIELE GmbH & Co. KG, Germany). Upper limit normal values were defined as ALT = 34 IU/L and AST = 31 IU/L for women and ALT = 45 IU/L and AST = 35 IU/L for men. Liver enzyme abnormalities were graded as follows: 1.25–2.5X the upper limit of normal (ULN) (grade 1), 2.6–5X ULN (grade 2), 5.1–10X ULN (grade 3), and >10X ULN (grade 4) [16]. Viral hepatitis (HBV and HCV) was screened using commercially available test kits for HBsAg (Ameritech-China, Ltd., USA) and for anti-HCV (Wondfo Biotech Co., Ltd., Guanfzhou, China).

To assure the quality of the data, the questionnaire was pretested. Two-day training was given to data collectors. Every day, the collected data were reviewed and checked for completeness. Standardized operating procedures and manufacturer's instructions were strictly followed for all laboratory procedures. Controls (Humatrol N and Humatrol P) were used for liver function tests in each test procedure. ELISA confirmed positive and negative samples were used in each test procedure for HBV and HCV testing kits performance checkup.

### 2.4. Data Processing and Analysis

The collected data were double-entered into EPI info version 7.0 to ensure data quality. The data were analyzed using SPSS version 20. Descriptive and summary statistics were calculated. ALT and AST enzyme levels were determined. The Mann Whitney *U* test and the independent *t*-test were used to compare nonparametric and parametric variables, respectively. Variables with *P* value ≤ 0.2 in the bivariate analysis were entered into multivariate analysis in backward LR method. *P* value < 0.05 in the multivariate analysis was taken as significant factor for elevated liver enzyme.

### 2.5. Ethical Consideration

Ethical clearance was obtained from University of Gondar School of Biomedical and Laboratory Sciences Ethical Review Committee. Official permission was obtained from Debre Tabor Hospital. Each study participant was informed about the purpose of the study and written informed consent was obtained from them. Results were kept confidential and abnormal results were reported to the physicians and professional nurses working at Debre Tabor Hospital HIV Clinic for management.

## 3. Results

### 3.1. Sociodemographic Characteristics

A total of 164 HAART experienced and 164 HAART naïve HIV infected patients participated in this study. The mean ages of the HAART experienced and HAART naïve groups were 36.29 ± 10.27 and 34.41 ± 10.73 years, respectively. Ninety (54.9%) HAART experienced and 111 (67.7%) HAART naïve patients were females. Most of the subjects were illiterate and married in 60 (36.6%) and 74 (45.1%) of HAART experienced groups, respectively (Table 1).

### 3.2. Clinical and Immunological Profile

The HAART experienced group had 135 (±91.35) cells/mm^3^ mean (±SD) baseline CD4 count and 41.38 (±21.58) months' mean (±SD) treatment duration. More than half of the patients were on 1a (52 [31.7%]) and 1c (40 [24.4%]) regimens. Majority of patients, 162 (98.8%), had good adherence to the regimens. In the HAART naïve group, 97 (51.1%) were under WHO clinical stage I and 12 (7.3%) were positive for HBV infection (Table 2). The HAART experienced group had significantly higher mean CD4 count compared to HAART naïve patients (HAART experienced: 356.88 cells/mm^3^; HAART naïve: 283.63 cells/mm^3^) (*P* < 0.0001).

### 3.3. Liver Enzyme Abnormalities

Thirty-three (20.1%) HAART experienced and 36 (22.0%) HAART naïve patients had elevated liver enzyme abnormalities in at least one biomarker (AST and/or ALT). ALT, AST, and both ALT and AST were elevated in 22 (13.4%), 25 (15.2%), and 14 (8.5%) of HAART experienced groups, respectively. In the HAART naïve groups, elevated ALT, AST, and both ALT and AST were found in 18 (11.0%), 29 (17.7%), and 10 (6.1%), respectively. Eleven (6.4%) HAART experienced and 3 (1.8%) HAART naïve patients had toxicity grade 2 or 3 in either abnormal ALT or AST. Hepatotoxicity grade 1 was found in 11 (6.7%) HAART experienced and 17 (10.4%) HAART naïve patients had grade 1 hepatotoxicity in ALT level (Table 2). The HAART experienced groups had significantly higher mean ALT level compared to HAART naïve groups (HAART experienced: 35 IU/L; HAART naïve: 24 IU/L; *P* = 0.002).

### 3.4. Factors for Liver Enzyme Abnormalities

Viral hepatitis (HBV and/or HCV), opportunistic infections, being male, and current CD4 count <200 cells/mm^3^ were significantly associated with elevated liver enzymes. Accordingly, HIV-1 patients with HBV and/or HCV infections were about 6 (AOR = 6.02; 95% CI = 1.87–19.39) times more likely to have elevated ALT/AST levels. Those patients with history of opportunistic infections were 2.91 (AOR = 2.91; 95% CI = 1.04–8.19) times more likely to have raised ALT/AST. Moreover, male HIV-1 patients had about 2 (AOR = 1.83; 95% CI = 1.001–3.33) times more elevated liver enzyme compared to females. Those patients with current CD4 count <200 cells/mm^3^ were 2.16 (AOR = 2.16; 95% CI = 1.06–4.39) times more likely to have raised liver enzymes as compared to those patients with CD4 count >350 cells/mm^3^ (Table 3).

## 4. Discussion

The prevalence of liver enzyme abnormalities in this study was 20.1% in HAART experienced and 22.0% in HAART naïve HIV patients. This finding is similar to other studies conducted on HIV infected patients in Cameroon (22.6%), South Africa (23%), and Brazil (19.7%) [17–19]. However, it was higher compared to the 11% prevalence in the general population of Australia [20]. The elevated liver enzyme in HIV infected patients might be due to direct inflammation of hepatocytes by HIV through apoptosis, mitochondrial dysfunction, and permeability alteration in mitochondrial membrane that stimulate an inflammatory response [12, 21–23].

Adverse drug reactions due to HAART are common. Its severity may range from mild to life-threatening conditions. They usually occur within the first 6–12 weeks but metabolic toxicities happen following prolonged use of antiretroviral therapy. Severe hepatotoxicity is a life-threatening condition (silent killer) that could affect patients on HAART. Patients with grade 3 hepatotoxicity should be immediately linked to ART physicians to substitute offending drug without stopping the HAART whereas HAART experienced patients with grade 4 hepatotoxicity should immediately discontinue all antiretroviral drugs and reintroduce modified regimen when the patient stabilized after supportive therapy [16]. In this study, 5 (3.1%) HAART experienced HIV infected patients had severe hepatotoxicity (Grade 3). Hence, regular monitoring of liver enzyme abnormalities is essential to manage such patients on HAART.

In the present study, HAART experienced HIV-1 patients had higher mean ALT (*P* value: 0.002). A recent study conducted by Owiredu et al. [24] has reported higher ALT level on HAART experienced HIV patients. The possible reason might be due to class-specific effects of drugs and cause direct mitochondrial toxicity of the liver as well as other organs that may lead to liver failure and lactic acidosis [25, 26].

In this study, current CD4 count <200 cells/mm^3^ was associated with elevated liver enzyme. Contradicting data were reported by different investigators [27, 28] which might be related to the fact that enhancement of immunity could be deleterious in diseases that involve immune-mediated mechanisms, such as autoimmune diseases or chronic viral hepatitis, or to the fact that enhanced immunity could also on the other hand decrease the opportunistic infection during HIV infection contributing to the reduction of liver enzymes.

In this study, we found significantly higher prevalence of elevated liver enzyme in HIV-1 patients coinfected with viral hepatitis (HBV and HCV). Previous studies also found significant association of viral hepatitis with hepatotoxicity [8, 29–31]. Viral cytopathic effect [32], raised HBV DNA viral load [33, 34], and mutation in the HBV precore and overlapping core genes (often associated with higher HBV DNA concentrations) [35] might contribute to elevated liver enzymes in those coinfected patients.

In this study, we found three times more elevated liver enzyme in male HIV-1 patients. Studies conducted in Italy [36], US community [37], and Thailand [38] reported similar association.

Immune abnormalities contribute to an increased risk of opportunistic infection among HIV infected patients. Although HIV infection is most closely associated with altered cell-mediated immunity, a number of additional immune deficiencies may occur in association with HIV infection. It usually includes a poor antibody response due to B cell dysfunction and defects in chemotaxis, phagocytosis, and intracellular killing by monocytes, macrophages, and neutrophils. Impairment of local defenses, manifested by a depression of specific IgA at the mucosal surfaces, also contributes to increased opportunistic infections [39]. In this study patients with history of opportunistic infections had significantly higher prevalence of elevated liver enzyme. Previous findings reported elevated liver enzyme in HIV patients with opportunistic infections [9, 40, 41]. This might be due to the fact that opportunistic infections such as viruses from the herpesviridae family (HSV, CMV), parasites (*Toxoplasma gondii*), mycobacteria (*M. tuberculosis*,* M. avium* complex), and fungi (*Cryptococcus*,* Histoplasma*) can all affect liver and manifest mostly as elevated liver enzymes [11].

*Limitations.* Patients with elevated liver enzyme were not confirmed by ultrasound whether their liver was really abnormal or not, a number of disease conditions were not fully addressed about whether they elevate liver enzyme, and HIV negative controls were not used due to budget constraint.

## 5. Conclusions

We found higher liver enzyme abnormalities in both HAART experienced and HAART naïve HIV-1 infected patients. The risk of developing liver enzyme abnormality was high among those with HBV and/or HCV infections, those with opportunistic infections, male patients, and those with low CD4 count. Therefore, monitoring and management of liver enzyme abnormalities in HIV infected patients are essential in our setting.

## Figures and Tables

**Table 1:** Sociodemographic characteristics of study participants, 2013.

Characteristics	HAART status	
	On HAART (%)	HAART naive (%)
Sex		
Male	74 (45.1)	53 (32.3)
Female	90 (54.9)	111 (67.7)

Age in years		
<25	12 (7.3)	32 (19.5)
25–34	59 (36.0)	58 (35.4)
35–44	59 (36.0)	43 (26.2)
>44	34 (20.7)	31 (18.9)

Education		
Illiterate	60 (36.6)	77 (47.0)
Elementary	52 (31.7)	41 (25.0)
High school	29 (17.7)	21 (12.8)
College & above	23 (14.0)	25 (15.2)

Marital status		
Single	32 (19.5)	30 (18.3)
Married	74 (45.1)	72 (43.9)
Divorced	29 (17.7)	39 (23.8)
Widowed	29 (17.7)	23 (14.0)

Residence		
Rural	32 (19.5)	50 (30.5)
Urban	132 (80.5)	114 (69.5)

HAART: highly active antiretroviral therapy.

**Table 2:** Clinical and immunological profile of HIV-1 patients at Debre Tabor Hospital, 2013.

Variables	HAART status	
	On HAART (%)	HAART naive (%)
WHO stage		
Stage I	34 (20.7)	97 (59.1)
Stage II	40 (24.4)	44 (26.8)
Stage III	64 (39.0)	19 (11.6)
Stage IV	26 (15.9)	4 (2.4)

OIs		
No	150 (91.5)	134 (81.7)
Yes	14 (8.5)	30 (18.3)

BMI (kg/m^2^)		
<18.5	39 (23.8)	40 (24.4)
18.5–25	113 (68.9)	116 (70.7)
>25	12 (7.3)	8 (4.9)

HBV		
Negative	156 (95.1)	152 (92.7)
Positive	8 (4.9)	12 (7.3)

HCV		
Negative	163 (99.4)	162 (98.8)
Positive	1 (0.6)	2 (1.2)

Regimen		
1a	52 (31.7)	—
1b	28 (17.1)	—
1c	40 (24.4)	—
1d	10 (6.1)	—
1e	18 (11.0)	—
1f	16 (9.8)	—

Hepatotoxicity		
Grade 1	11 (6.7)	17 (10.4)
Grade 2	6 (3.7)	0
Grade 3	5 (3.0)	1 (0.6)

WHO: World Health Organization; OI: opportunistic infection; BMI: body mass index; HBV: hepatitis B virus; HCV: hepatitis C virus; HAART: highly active antiretroviral therapy; 1a: d4T+3TC+NVP; 1b: d4T+3TC+ EFV; 1c: AZT+3TC+ NVP; 1d: AZT+3TC+EFV; 1e: TDF+3TC+EFV; 1f: TDF+3TC+NVP.

**Table 3:** Multiple logistic regression analysis of factors for ALT and/or AST enzyme abnormalities in HIV-1 infected patients at Debre Tabor Hospital, 2013.

Variables	Liver enzyme		COR (95% CI)	AOR (95% CI)
	Normal (%)	Abnormal (%)		
Education level				
Illiterate	130 (42.8)	7 (29.2)	0.59 (0.17–2.12)	0.52 (0.14–1.97)
Elementary	82 (27.0)	11 (45.8)	1.48 (0.44–4.91)	1.54 (0.44–5.41)
High school	48 (15.8)	2 (8.3)	0.46 (0.08–2.63)	0.34 (0.06–2.078)
College & above	44 (14.5)	4 (16.7)	1	1

Marital status				
Single	53 (17.4)	9 (37.5)	1	—
Married	136 (44.7)	10 (41.7)	0.43 (0.17–1.13)	—
Divorced	66 (21.7)	2 (8.3)	0.18 (0.04–0.86)	—
Widowed	49 (16.1)	3 (12.5)	0.36 (0.09–1.41)	—

Sex				
Male	100 (36.5)	27 (50.0)	1.74 (0.98–3.13)	1.83 (1.001–3.33)^*∗*^
Female	174 (63.5)	27 (50.0)	1	1

CD4 count/cells/mm^3^				
<200	73 (26.6)	24 (44.4)	2.10 (1.04–4.22)	2.16 (1.06–4.39)^*∗*^
200–350	99 (36.1)	14 (25.9)	0.90 (0.42–1.95)	0.91 (0.42–1.98)
>350	102 (37.2)	16 (29.6)	1	1

OIs				
No	266 (87.5)	18 (75.0)	1	1
Yes	38 (12.5)	6 (25.0)	2.33 (0.87–6.25)	2.91 (1.04–8.19)^*∗*^

HAART status				
No	154 (50.7)	10 (41.7)	1	—
Yes	150 (49.3)	14 (58.3)	1.44 (0.62–3.34)	—

HBV or HCV				
Positive	17 (5.6)	5 (20.8)	4.44 (1.48–13.35)	6.02 (1.87–19.39)^*∗*^
Negative	287 (94.4)	19 (79.2)	1	1

^*∗*^Significantly associated; OI: opportunistic infection; HBV: hepatitis B virus; HCV: hepatitis C virus; HAART: highly active antiretroviral therapy; AOR: adjusted odds ratio; COR: crude odds ratio; CI: confidence interval.

## Abbreviations

AIDS: Acquired Immunodeficiency Disease Syndrome
ALT: Alanine aminotransferase
AST: Aspartate aminotransferase
HBsAg: Hepatitis B surface antigen
HBV: Hepatitis B virus
HCV: Hepatitis C virus
HIV-1: Human Immunodeficiency Virus 1.

## References

[1] Palella F. J., Baker R. K., Moorman A. C. (2006). Mortality in the highly active antiretroviral therapy era: changing causes of death and disease in the HIV outpatient study. *Journal of Acquired Immune Deficiency Syndromes*.

[2] Smith C., Sabin C. A., Lundgren J. D. (2010). Factors associated with specific causes of death amongst HIV-positive individuals in the D:A:D Study. *AIDS*.

[3] Bica I., McGovern B., Dhar R. (2001). Increasing mortality due to end-stage liver disease in patients with human immunodeficiency virus infection. *Clinical Infectious Diseases*.

[4] Martín-Carbonero L., Soriano V., Valencia E., García-Samaniego J., López M., González-Lahoz J. (2001). Increasing impact of chronic viral hepatitis on hospital admissions and mortality among HIV-infected patients. *AIDS Research and Human Retroviruses*.

[5] Castellares C., Barreiro P., Martín-Carbonero L. (2008). Liver cirrhosis in HIV-infected patients: prevalence, aetiology and clinical outcome. *Journal of Viral Hepatitis*.

[6] Richardson J., Melester D. (2003). Treatment AIDS. *Clinical Liver Disease*.

[7] Ogedegbe A. O., Sulkowski M. S. (2003). Antiretroviral-associated liver injury. *Clinics in Liver Disease*.

[8] Núñez M., Lana R., Mendoza J. L., Martín-Carbonero L., Soriano V. (2001). Risk factors for severe hepatic injury after introduction of highly active antiretroviral therapy. *Journal of Acquired Immune Deficiency Syndromes*.

[9] Cappell M. S. (1991). Hepatobiliary manifestations of the acquired immune deficiency syndrome. *The American Journal of Gastroenterology*.

[10] Lefkowitch J. H. (1994). Pathology of AIDS-related liver disease. *Digestive Diseases*.

[11] Butt A. (2012). *Epidemiology of Liver Disease in Human Immunodeficiency Virus-Infected Persons*.

[12] Pol S., Lebray P., Vallet-Pichard A. (2004). HIV infection and hepatic enzyme abnormalities: intricacies of the pathogenic mechanisms. *Clinical Infectious Diseases*.

[13] Sulkowski M. S. (2008). Management of hepatic complications in HIV-infected persons. *Journal of Infectious Diseases*.

[14] Crum-Cianflone N., Collins G., Medina S. (2010). Prevalence and factors associated with liver test abnormalities among human immunodeficiency virus-infected persons. *Clinical Gastroenterology and Hepatology*.

[15] Ocama P., Katwere M., Piloya T. (2008). The spectrum of liver diseases in HIV infected individuals at an HIV treatment clinic in Kampala, Uganda. *African Health Sciences*.

[16] Federal HIV/AIDS Prevention and Control Office http://www.who.int/hiv/pub/guidelines/ethiopia_art.pdf.

[17] Gil A. C. M., Lorenzetti R., Mendes G. B. (2007). Hepatotoxicity in HIV-infected children and adolescents on antiretroviral therapy. *Sao Paulo Medical Journal*.

[18] Hoffmann C. J., Charalambous S., Thio C. L. (2007). Hepatotoxicity in an African antiretroviral therapy cohort: the effect of tuberculosis and hepatitis B. *AIDS*.

[19] Lucent K., Clement A., Fon N., Weldeji P., Ndikvu C. (2010). The effects of antiretroviral treatment on liver function enzymes among HIV infected out patients attending the central hospital of Yaoundé Cameron. *African Journal of Clinical and Experimental Microbiology*.

[20] Australian Bureau of Statistics (2013). *Australian Health Survey: Biomedical Results for Chronic Diseases, 2011-12*.

[21] Côté H., Brumme Z. L., Craib K. J. (2002). Changes in mitocondrial DNA as a marker of nucleoside toxicity in HIV-infected patients. *The New England Journal of Medicine*.

[22] Jacotot E., Ravagnan L., Loeffler M. (2000). The HIV-1 viral protein R induces apoptosis via a direct effect on the mitochondrial permeability transition pore. *Journal of Experimental Medicine*.

[23] Gross A., Jockel J., Wei M. C., Korsmeyer S. J. (1998). Enforced dimerization of BAX results in its translocation, mitochondrial dysfunction and apoptosis. *The EMBO Journal*.

[24] Owiredu W. K., Quaye L., Amidu N. (2011). Oxidative stress and Dyslipidaemia among Ghanaian HAART-naïve HIV patients and those on HAART. *West African Journal of Pharmacy*.

[25] Sulkowski M., Thomas D., Mehta S., Chaisson R., Moore R. (2003). Hepatotoxicity associated with the antiretroviral therapy containing protease inhibitors with or without pharmacokinetic boosting by low-dose ritonavir. *Hepatology*.

[26] Murphy R. A., Sunpath H., Kuritzkes D. R., Venter F., Gandhi R. T. (2007). Antiretroviral therapy-associated toxicities in the resource-poor world: the challenge of a limited formulary. *Journal of Infectious Diseases*.

[27] Ballardini G., Groff P., Pontisso P. (1995). Hepatitis C virus genotype, tissue HCV antigens, hepatocellular expression of HLA-A, B, C and intercellular adhesion molecules: clues to pathogenesis of hepatocellular damage and response to interferon treatment in patients with chronic hepatitis C. *The Journal of Clinical Investigation*.

[28] Nelson D. R., Marousis C. G., Davis G. L. (1997). The role of hepatitis C virus-specific cytotoxic T lymphocytes in chronic hepatitis C. *Journal of Immunology*.

[29] Ena J., Amador C., Benito C., Fenoll V., Pasquau F. (2003). Risk and determinants of developing severe liver toxicity during therapy with nevirapine-and efavirenz-containing regimens in HIV-infected patients. *International Journal of STD & AIDS*.

[30] Thio C. L., Smeaton L., Saulynas M. (2013). Characterization of HIV-HBV coinfection in a multinational HIV-infected cohort. *AIDS*.

[31] Audsley J., Seaberg E. C., Sasadeusz J. (2011). Factors associated with elevated ALT in an international HIV/HBV co-infected cohort on long-term HAART. *PLoS ONE*.

[32] Fang J. S., Wright T., Lau J. N. (1993). Fibrosing cholestatic hepatitis in patient with HIV and hepatitis B. *The Lancet*.

[33] Thio C. L., Seaberg E. C., Skolasky R. (2002). HIV-1, hepatitis B virus, and risk of liver-related mortality in the Multicenter Cohort Study (MACS). *The Lancet*.

[34] Bräu N., Fox R. K., Xiao P. (2007). Presentation and outcome of hepatocellular carcinoma in HIV-infected patients: a U.S.-Canadian multicenter study. *Journal of Hepatology*.

[35] Revill P. A., Littlejohn M., Ayres A. (2007). Identification of a novel hepatitis B virus precore/core deletion mutant in HIV/hepatitis B virus co-infected individuals. *AIDS*.

[36] Guaraldi G., Squillace N., Stentarelli C. (2008). Nonalcoholic fatty liver disease in HIV-infected patients referred to a metabolic clinic: prevalence, characteristics, and predictors. *Clinical Infectious Diseases*.

[37] Kim W. R., Benson J. T., Therneau T. M., Torgerson H. A., Yawn B. P., Melton L. J. (2004). Changing epidemiology of hepatitis B in a U.S. Community. *Hepatology*.

[38] Chalermchai T., Hiransuthikul N., Tangkijvanich P., Pinyakorn S., Avihingsanon A., Ananworanich J. (2013). Risk factors of chronic hepatitis in antiretroviral-treated HIV infection, without hepatitis B or C viral infection. *AIDS Research and Therapy*.

[39] Çiledağ A., Karnak D. (2011). *AIDS and Opportunistic Infections*.

[40] Al Anazi A. R. (2009). Gastrointestinal opportunistic infections in human immunodeficiency virus disease. *Saudi Journal of Gastroenterology*.

[41] Terzic D., Brmbolic B., Jevtovic D. (2008). Liver enlargement associated with opportunistic infections in patients with human immunodeficiency virus infection. *Journal of Gastrointestinal and Liver Diseases*.

